# PATIENT-REPORTED PAIN AND SLEEP DISTURBANCE AFTER HOSPITALIZATION FOR MEDICAL ILLNESS

**DOI:** 10.1093/geroni/igae098.0717

**Published:** 2024-12-31

**Authors:** Susan Hastings, Karen Stechuchak, Cynthia Coffman, Caitlin Kappler, Jaime Hughes, Amy Webster, Kelli Allen

**Affiliations:** Durham VA, Durham, North Carolina, United States; Durham VA Health Care System, Durham, North Carolina, United States; Durham VA Health Care System, Durham, North Carolina, United States; Durham VA Health Care System, Durham, North Carolina, United States; Wake Forest University School of Medicine, Winston-Salem, North Carolina, United States; Durham VA Health Care, Durham, North Carolina, United States; Durham VA Health Care System, Durham, North Carolina, United States

## Abstract

Older adults experience a well-documented period of vulnerability following hospitalization; yet contributing factors are not fully understood. The goal of this cross-sectional study was to examine associations between post-discharge pain and sleep disturbance with quality of life (QoL) and function in the month following hospitalization for medical illness. As part of a larger study evaluating the effectiveness and implementation of a hospital-based mobility program, we conducted telephone surveys with 618 adults aged ≥60 discharged from one of 8 Veterans Affairs (VA) hospitals. Primary predictors of interest were pain interference and sleep disturbance measured by PROMIS-29 short forms. Outcomes were QoL measured by EQ-5D, function measured by late-life function and disability instruments (LLFDI total and advanced lower extremity function) and community mobility measured by Life Space. For each outcome, separate multiple linear regression models were fit including the key explanatory variables of interest (pain interference and sleep disturbance) and selected covariates (sociodemographic, clinical, hospitalization characteristics) social/regional factors (e.g., social vulnerability, hospital). Participants were mean age 70.6 years, 95% male, 25% Black, 34% lived alone, 8% reported being discharged to a nursing home and 15% had been readmitted to the hospital. In adjusted models, pain interference and sleep disturbance were negatively associated with QoL, total function, lower extremity function, and community mobility (P-values < 0.01). In this sample of recently hospitalized older adults, pain and sleep disturbance were independent contributors to worse QoL and functional status. Pain and sleep may warrant further attention and/or intervention during the post-hospitalization period.

